# The expression of NOD2, NLRP3 and NLRC5 and renal injury in anti-neutrophil cytoplasmic antibody-associated vasculitis

**DOI:** 10.1186/s12967-019-1949-5

**Published:** 2019-06-11

**Authors:** Luo-Yi Wang, Xiao-Jing Sun, Min Chen, Ming-Hui Zhao

**Affiliations:** 1 Renal Division, Department of Medicine, Peking University First Hospital, Peking University Institute of Nephrology, No 8, Xishiku Street, Xicheng District, Beijing, 100034 China; 2 Key Laboratory of Renal Disease, Ministry of Health of China, Beijing, China; 3 Key Laboratory of Chronic Kidney Disease Prevention and Treatment (Peking University), Ministry of Education, Beijing, China; 4 Peking-Tsinghua Center for Life Sciences, Beijing, China

**Keywords:** ANCA, NOD-like receptors, Vasculitis

## Abstract

**Background:**

Nucleotide-binding oligomerization domain (NOD)-like receptors (NLRs) are intracellular sensors of pathogens and molecules from damaged cells to regulate the inflammatory response in the innate immune system. Emerging evidences suggested a potential role of NLRs in anti-neutrophil cytoplasmic antibody (ANCA)-associated vasculitis (AAV). This study aimed to investigate the expression of nucleotide-binding oligomerization domain containing protein 2 (NOD2), NOD-like receptor family pyrin domain containing 3 (NLRP3) and NOD-like receptor family CARD domain containing 5 (NLRC5) in kidneys of AAV patients, and further explored their associations with clinical and pathological parameters.

**Methods:**

Thirty-four AAV patients in active stage were recruited. Their renal specimens were processed with immunohistochemistry to assess the expression of three NLRs, and with double immunofluorescence to detect NLRs on intrinsic and infiltrating cells. Analysis of gene expression was also adopted in cultured human podocytes. The associations between expression of NLRs and clinicopathological parameters were analyzed.

**Results:**

The expression of NOD2, NLRP3 and NLRC5 was significantly higher in kidneys from AAV patients than those from normal controls, minimal change disease or class IV lupus nephritis. These NLRs co-localized with podocytes and infiltrating inflammatory cells. The mean optical density of NOD2 in glomeruli was significantly higher in crescentic class than non-crescentic class, and correlated with levels of proteinuria and serum creatinine at renal biopsy. The mean optical density of NLRC5 in glomeruli was significantly higher in crescentic class than non-crescentic class, and correlated with proteinuria level, Birmingham Vasculitis Activity Score and the proportion of crescents in the renal specimen.

**Conclusions:**

The expression of three NLRs was upregulated in kidneys of AAV patients. The expression of NOD2 and NLRC5 was associated with the severity of renal lesions in AAV.

## Background

Anti-neutrophil cytoplasmic antibody (ANCA)-associated vasculitis (AAV) is a group of autoimmune diseases, characterized by pauci-immune necrotizing crescentic glomerulonephritis in renal histology. AAV includes granulomatosis with polyangiitis (GPA), microscopic polyangiitis (MPA) and eosinophilic granulomatosis with polyangiitis (EGPA). ANCAs specific for myeloperoxidase (MPO) or proteinase 3 (PR3) are main pathogenic autoantibodies in AAV [1].

Accumulating evidences suggest that many constituents of innate immune system, such as the complement system, play important roles in the pathogenesis of AAV [2–4]. The pattern recognition receptor is another critical component of the innate immune system as sensors of exogenous pathogens and harmful molecules [5]. As one of the most important components of the germline-encoded recognition system, nucleotide-binding oligomerization domain (NOD)-like receptors (NLRs) participate in a number of infectious diseases, autoimmune diseases and kidney diseases [5–12]. Among numerous NLRs, The classic antimicrobic receptor nucleotide-binding oligomerization domain containing protein 2 (NOD2) showed enhanced pro-inflammatory effects with PR3-ANCA in vitro [13, 14], and has been reported to mediate the hyperglycemia-induced podocyte dysfunction in diabetic nephropathy [15]. NOD-like receptor family pyrin domain containing 3 (NLRP3) can mediate the assembly of a cytosolic multiprotein complex called inflammasome, and the subsequent IL-1β and IL-18 might be involved in AAV [16–19]. Besides NOD2 and NLRP3, NOD-like receptor family CARD domain containing 5 (NLRC5), the largest NLR, also participates in the developments of many kidney diseases including ischemia–reperfusion injury and diabetic nephropathy in various manners [20–24]. However, the role of NLRs remains largely unknown in AAV. The aim of this study was to investigate the expression of NOD2, NLRP3 and NLRC5 in kidneys of AAV patients, and further explore their association with clinical and pathological parameters.

## Methods

### Patients and specimens

Thirty-four patients with active AAV receiving renal biopsies before immunosuppressive therapy in Peking University First Hospital from 2016 to 2018 were enrolled in this study. All the patients met the criteria of the 2012 Chapel Hill Consensus Conference definition for AAV [1]. No patient had coexistence of other kidney diseases, secondary vasculitis, other autoimmune diseases or infections at the time of renal biopsy according to the clinical and laboratory data. Renal tissue from normal parts of nephrectomized kidneys from seventeen patients with solitary renal cell carcinoma was collected as normal controls. They were identified normal with regular light microscopy, immunofluorescence and electron microscopy. Renal specimens from nineteen patients with minimal change disease (MCD) and twenty patients with class IV lupus nephritis (LN) according to the International Society of Nephrology/Renal Pathology Society (ISN/RPS) [25] were collected as disease controls. Informed consent was signed by each participant. This research was in compliance with the Declaration of Helsinki and approved by the ethics committee of Peking University First Hospital.

### Renal histopathology

Renal histopathology of AAV patients was evaluated according to the standardized protocols [26–28] described previously. In brief, the lesions of glomeruli including fibrinoid necrosis, glomerulosclerosis and total crescents were calculated as affected percentage of the total number of glomeruli in biopsies. The lesions of tubulointerstitium were semi-quantitatively scored according to the affected area of tubulointerstitium: tubular atrophy, interstitial fibrosis (− for 0%, + for 0–50% and ++  for > 50%) and interstitial infiltration (− for 0%, + for 0–25%, ++  for 25–50% and +++ for > 50%). Furthermore, each biopsy was classified as focal, crescentic, mixed or sclerotic category, according to the pathologic classification system for ANCA-associated glomerulonephritis proposed by Berden et al. [29].

### Assessment of NLR expression in kidneys with immunohistochemistry

Formaldehyde-fixed renal slides of AAV patients, normal and disease controls were dewaxed in xylene ethanol at room temperature and rehydration through graded ethanol. Antigen retrieval was then performed by heating the slides in citrate buffer (0.01 M, pH 6.0) in a pressure cooker at the highest pressure for 2 min and 30 s. After cooled to room temperature and washed with PBS, the slides were immersed in freshly prepared 3% hydrogen peroxide for 10 min at room temperature to quench endogenous peroxidase activity. Non-specific staining was blocked by incubating specimens with 3% bovine serum albumin (BSA) in PBS at room temperature for 60 min. After the removal of blocking BSA without washing, primary antibodies for NOD2 (1:500, 20,980–1-AP; ProteinTech Group, Chicago, USA), NLRP3 (1:200, ab214185; Abcam, Cambridge, UK) or NLRC5 (1:500, ab105411; Abcam, Cambridge, UK) were added and incubated overnight at 4 °C. For negative controls, specimens were incubated with 3% BSA instead of primary antibodies. Secondary antibodies (PV9001, ZSGB-Bio, Beijing, China) were incubated with the specimens at 37 °C for 20 min. Sections were developed in fresh hydrogen peroxide plus 3–3′-diaminobenzidine tetra hydrochloride solution for 40 s. Nuclear staining was performed by incubating the specimens with haematoxylin for 8 min. Finally, the slides were dehydrated through ethanol and xylene and sealed with neutral gum. All the glomeruli in a section at × 400 and at least 10 fields of tubulointerstitial vision per kidney section at × 400 were observed blindly for quantitative assessments of immunohistochemical staining.

The staining results of NLRs were evaluated by the Image Pro Plus (version 6.0; Media Cybernetics, Dallas, TX, USA): The optical intensity threshold of pictures was corrected to 0–255. Hue-Saturation-Intensity model was used to select stained area and calculate the integrated optical density and relative area in pixel. The mean optical density = integrated optical density/relative area.

### Detection of NLR expression on various cell types

We performed double immunofluorescence of NLRs and specific markers of various cell types, including renal intrinsic and inflammatory cells in AAV patients, to determine concrete locations of NLRs in the kidney from AAV patients. Glomerular endothelial cells, mesangial cells and podocytes were identified using immunofluorescence staining with primary antibodies against CD31 (1:50, sc53411; Santa Cruz Biotechnology, CA), integrin-α8 (1:50, sc365798; Santa Cruz Biotechnology, CA) and synaptopodin (1:50, MAB8977; R&D Systems, Minneapolis, MN, USA) respectively. Infiltrating cells (monocytes/macrophages) were identified using immunofluorescence staining with antibodies against CD68 (1:100, ab31630; Abcam, Cambridge, UK). After dewaxed, antigen retrieval and non-specific staining blocked, the renal specimens were incubated with mixed primary antibodies overnight at 4 °C and secondary antibody of Alexa Fluor (AF488)-labeled donkey anti-rabbit IgG (1:200; Jackson ImmunoResearch, West Grove, PA, USA) and cyanin 3 (Cy3)-labeled donkey anti-mouse IgG (1:200; Jackson ImmunoResearch) at 37 °C for 1 h. After washed with PBS, the specimens were stained with 4,6-diamidino-2-phenylindole (DAPI) and eventually mounted with Mowiol. For negative controls, the mixture of primary antibodies was replaced with 3% BSA. Confocal images were captured with a Zeiss LSM 710 confocal microscope (Zeiss, Jena, Germany). Images were exported from the ZEN 2012 (blue edition) microscopy software.

### Cell culture and treatments

The immortalized human podocyte cell line was a gift from Prof. Saleem MA. Immortalized human podocytes were grown at 33 °C in RPMI 1640 media supplemented with 10% fetal bovine serum (FBS, Gibco, Thermo Fisher Scientific, MA, USA) and insulin, transferrin, selenium, ethanolamine solution (ITS, 41400045, Thermo Fisher Scientific, MA, USA). To allow the cells to differentiate, the cells were grown to 70–80% confluence, trypsinized and transferred at a dilution of 1:4 in growth media and cultured at the non-permissive temperature of 37 °C (in 5% CO_2_). After 14 days, the differentiated podocytes were starved for 12 h followed by stimulation with 10 ng/ml human tumor necrosis factor α (TNF-α, R&D Systems, Minneapolis, MA, USA) for 12 h to imitate the inflammatory condition in AAV [30, 31].

### Quantitative polymerase chain reaction of NLRs

To determine messenger RNA (mRNA) expression of three NLRs, total RNA was extracted from differentiated podocytes using Trizol. After reverse transcription of RNA, the mRNA level of each target gene was quantified on ViiATM7 Dx Real-Time PCR (Applied Biosystems, Foster City, CA, USA) using SYBR Green Universal PCR Master Mix (Applied Biosystems) according to the manufacturer’s instructions. The fold change of expression levels were calculated using the comparative ΔCt method. Primers used for PCR: NOD2 forward ACCTTTGATGGCTTTGACG and reverse CACCTTGCGGGCATTCTT, NLRP3 forward TGAAGAAAGATTACCGTAAGAAGTACAGA and reverse GCGTTTGTTGAGGCTCACACT, NLRC5 forward TGGGAAGACACTCAGGCTAA and reverse ATCATCGTCCTCACAGAGGTT, beta-actin (housekeeping gene) forward ACCACACCTTCTACAATGAGC and reverse CAGCCTGGATAGCAACGTAC.

### Statistical analysis

Experimental statistics are shown as mean ± standard error for variables with Gaussian distribution and median (interquartile range, IQR) for the others. Differences of quantitative parameters among groups were assessed with *t-*test or one-way ANOVA for two or more independent samples as appropriated. Correlations among parametric variables were performed using Pearson's test while the correlations among one or more non-parametric variables were performed using Spearman’s test. Results were considered significant at *P* < 0.05. Data analysis was performed with SPSS version 20.0 (SPSS, Chicago, IL, USA).

## Results

### General data of AAV patients

Among the thirty-four patients with active ANCA-associated vasculitis, twelve were males and twenty-two were females with an age of 61.7 ± 11.9 years at renal biopsy. Thirty patients were MPO-ANCA positive and four patients were PR3-ANCA positive. The median level of serum creatinine at renal biopsy was 266.9 (IQR: 196.3–410.0) μmol/L. The Birmingham Vasculitis Activity Score (BVAS) [32] was 18.6 ± 5.2. The majority of histopathologic categorization was crescentic class (24/34, 70.6%), according to the pathologic classification system by Berden et al. [29]. Detailed data are listed in Table 1.

**Table 1:** General data of the AAV patients

Parameters	Value
Number	34
Gender (male/female)	12/22
Age at diagnosis	61.7 ± 11.9
MPO-ANCA/PR3-ANCA	30/4
ESR (mm/1 h)	65.8 ± 39.2
Scr (μmol/L)	266.9 (196.3–410.0)
Proteinuria (g/24 h)	1.42 ± 1.25
Clinical manifestation	
Skin rash	5 (14.7%)
Arthralgia	3 (8.8%)
Muscle pain	5 (14.7%)
Pulmonary	18 (52.9%)
ENT	10 (29.4%)
Ophthalmic	10 (29.4%)
Gastrointestinal	3 (8.8%)
Nervous	5 (14.7%)
BVAS	18.6 ± 5.2
Glomeruli per biopsy	30.0 ± 13.8
Glomerular lesions (%)	
Total crescents	62.2 ± 21.0
Fibrinoid necrosis	1.3 (0–6.6)
Glomerulosclerosis	2.7 (0–10.0)
Tubulointerstitial lesions	
Tubular atrophy (−/+/++)	5/25/4
Interstitial infiltration (−/+/++/+++ )	0/8/21/5
Interstitial fibrosis (−/+/++)	3/25/6
Berden classification n (%)	
Focal	5 (14.7)
Crescentic	24 (70.6)
Mixed	5 (14.7)
Sclerotic	0 (0)

Statistics are shown as mean ± standard deviation or median (IQR) for different distributions

*ESR* erythrocyte sedimentation rate, *Scr* serum creatinine, *ENT* ear, nose and throat, *BVAS* Birmingham Vasculitis Activity Score

### Intensities of NLRs expression in AAV patients

**Fig. 1:**
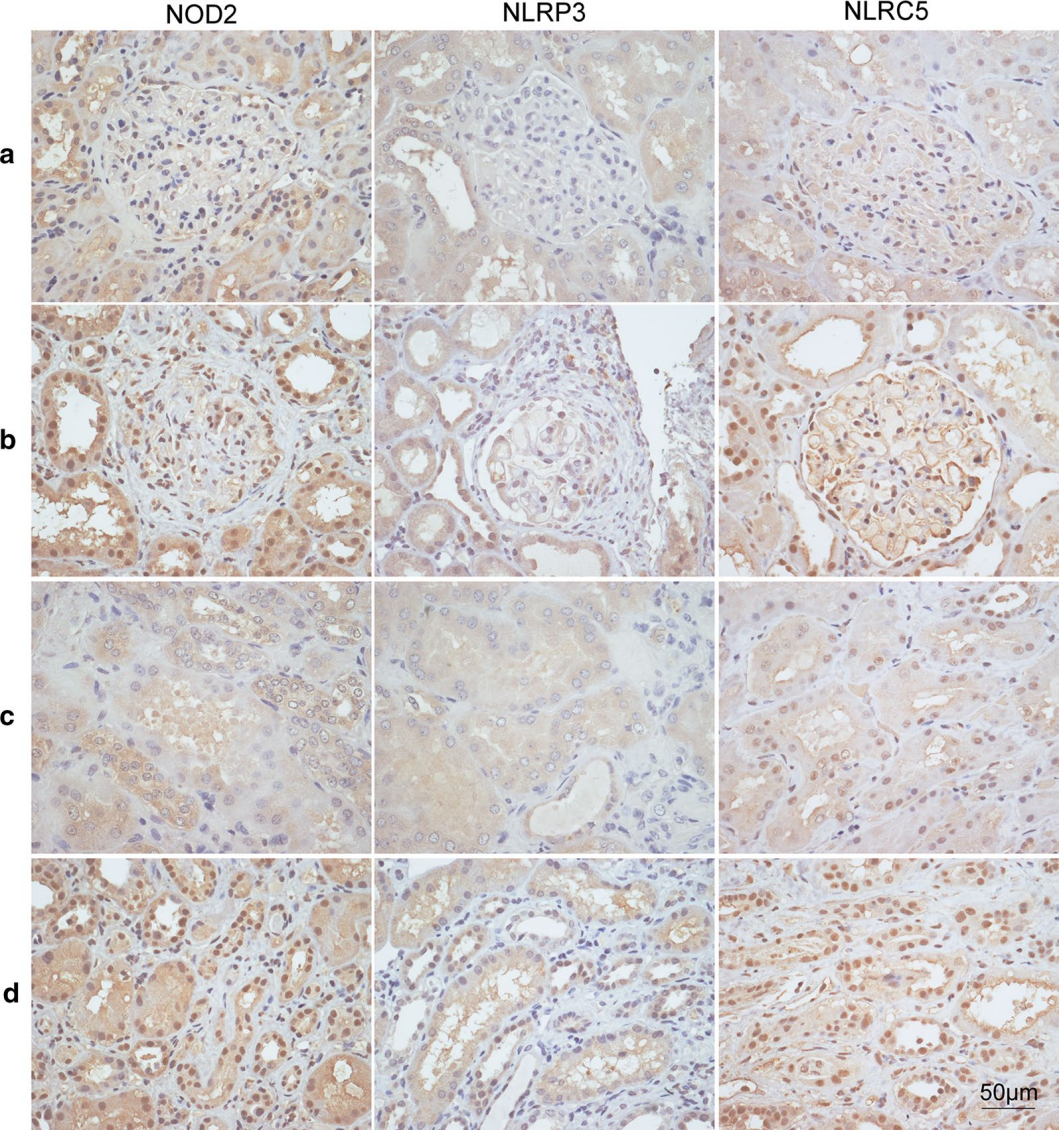
Immunohistochemistry staining for NOD2, NLRP3, NLRC5 in glomeruli and tubulointerstitium of renal specimens. **a** Immunohistochemical staining of NLRs in glomeruli of normal controls. **b** Immunohistochemical staining of NLRs in glomeruli of AAV patients. **c** Immunohistochemical staining of NLRs in tubulointerstitium of normal controls. **d** Immunohistochemical staining of NLRs in tubulointerstitium of AAV patients. Scale bar = 50 μm in the bottom right

In AAV patients, immunohistochemical staining showed that NOD2, NLRP3 and NLRC5 were widely expressed in glomeruli and tubulointerstitium (Fig. 1).

**Fig. 2:**
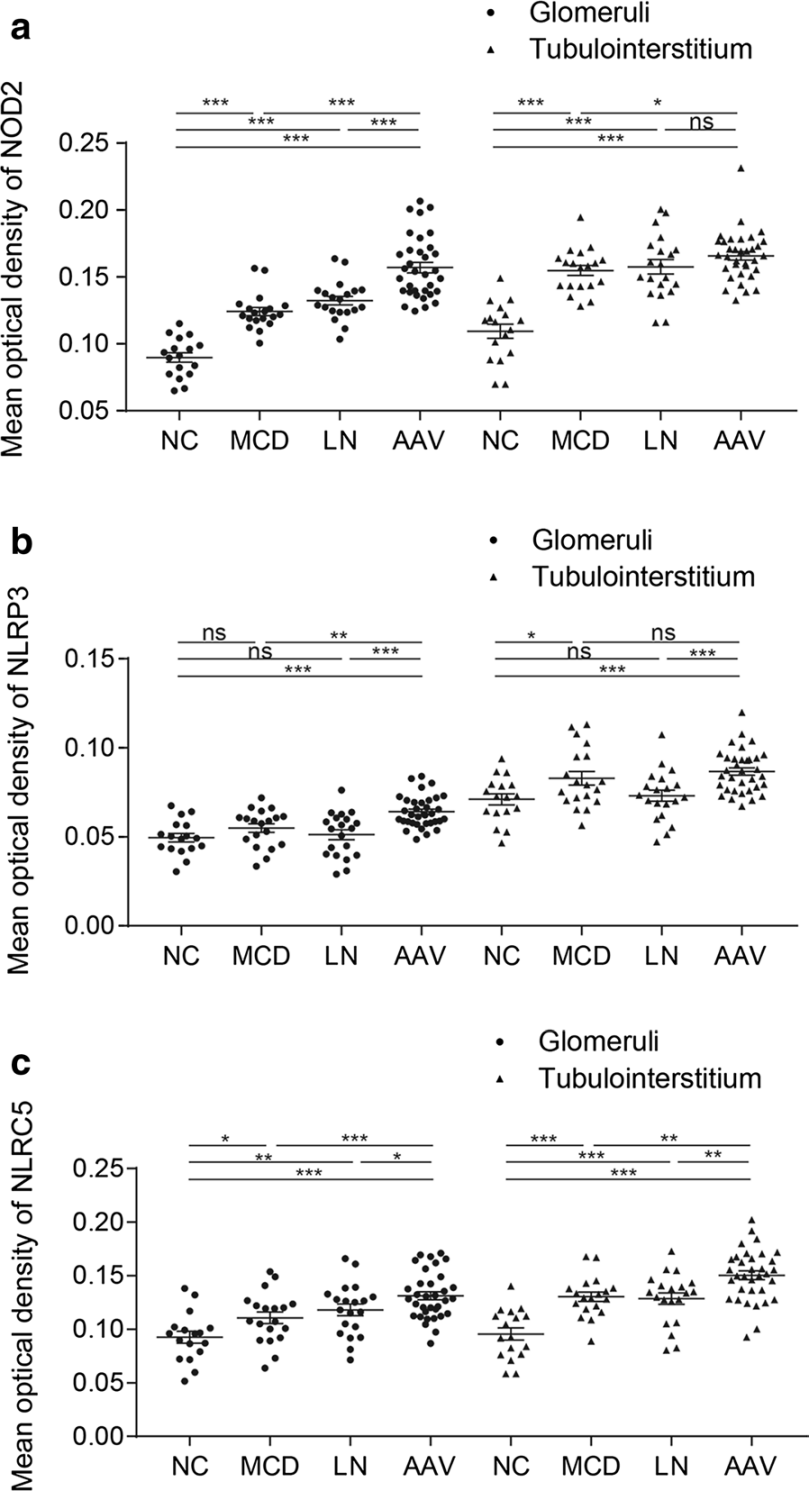
Different expression of NOD2, NLRP3, NLRC5. **a** The mean optical density of NOD2 in different conditions. **b** The mean optical density of NLRP3 in different conditions. **c** The mean optical density of NLRC5 in different conditions. NC: normal control. MCD: minimal change disease. *LN* lupus nephritis, *AAV* anti-neutrophil cytoplasmic antibody-associated vasculitis. Data were shown as mean ± standard error. *ns* no significance. *P < 0.05, **P < 0.01, ***P < 0.001

The mean optical densities of NOD2, NLRP3 and NLRC5 in glomeruli of AAV patients were significantly higher than those of normal controls (0.157 ± 0.004 vs. 0.090 ± 0.004, *P* < 0.001; 0.064 ± 0.002 vs. 0.050 ± 0.002, *P* < 0.001; 0.131 ± 0.004 vs. 0.093 ± 0.006, *P* < 0.001, respectively). Consistently, the mean optical densities of NOD2, NLRP3 and NLRC5 in tubulointerstitium were also significantly higher in AAV patients than those of normal controls (0.166 ± 0.003 vs. 0.109 ± 0.005, *P* < 0.001; 0.087 ± 0.002 vs. 0.071 ± 0.003, *P* < 0.001; 0.150 ± 0.004 vs*.* 0.096 ± 0.006, *P* < 0.001, respectively). Compared with disease controls, the expression of three NLRs in AAV patients was significantly higher than those in MCD or class IV LN. There were also close correlations between the expression in glomeruli and tubulointerstitium in AAV patients (r = 0.644, *P* < 0.001 for NOD2; r = 0.762, *P* < 0.001 for NLRP3; r = 0.844, *P* < 0.001 for NLRC5) (Fig. 2).

### Co-localization of NLRs with renal intrinsic cells and infiltrating cells

**Fig. 3:**
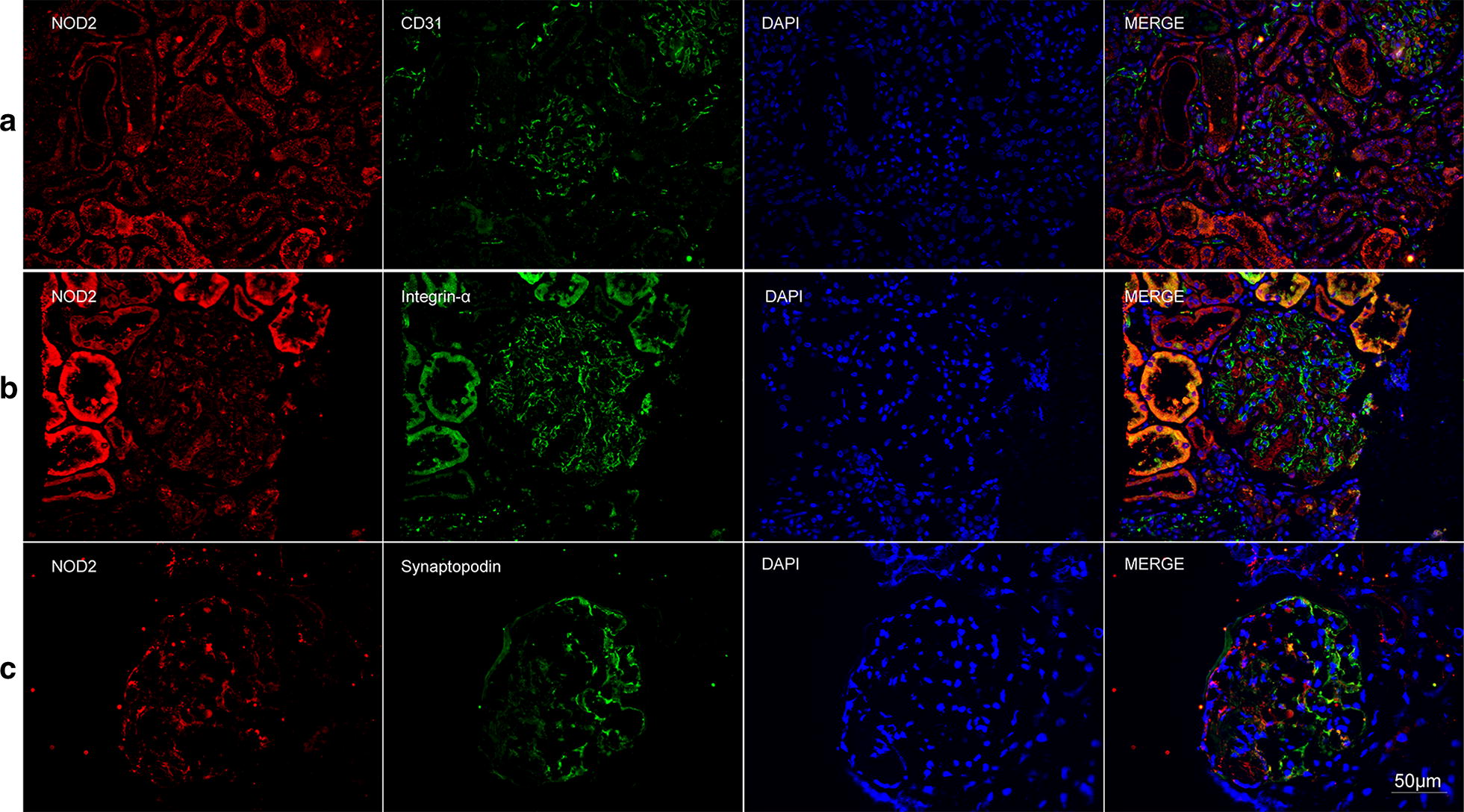
Double immunofluorescence staining of NOD2 and glomerular intrinsic cells in AAV patients. **a** Co-localization of NOD2 (red) and CD31 (green). **b** Co-localization of NOD2 (red) and integrin-α (green). **c** Co-localization of NOD2 (red) and synaptopodin (green). Scale bar = 50 μm in the bottom right

**Fig. 4:**
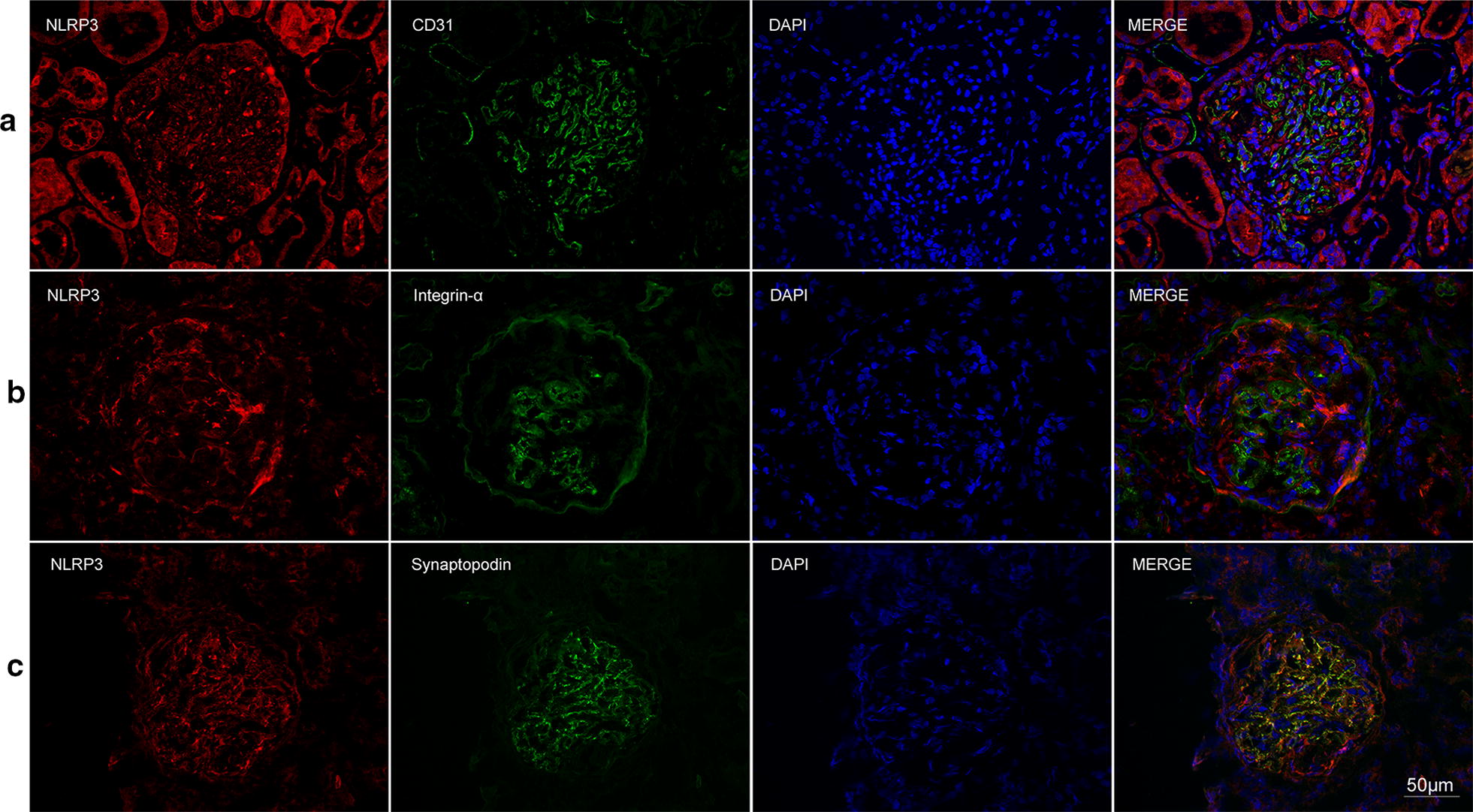
Double immunofluorescence staining of NLRP3 and glomerular intrinsic cells in AAV patients. **a** Co-localization of NLRP3 (red) and CD31 (green). **b** Co-localization of NLRP3 (red) and integrin-α (green). **c** Co-localization of NLRP3 (red) and synaptopodin (green). Scale bar = 50 μm in the bottom right

**Fig. 5:**
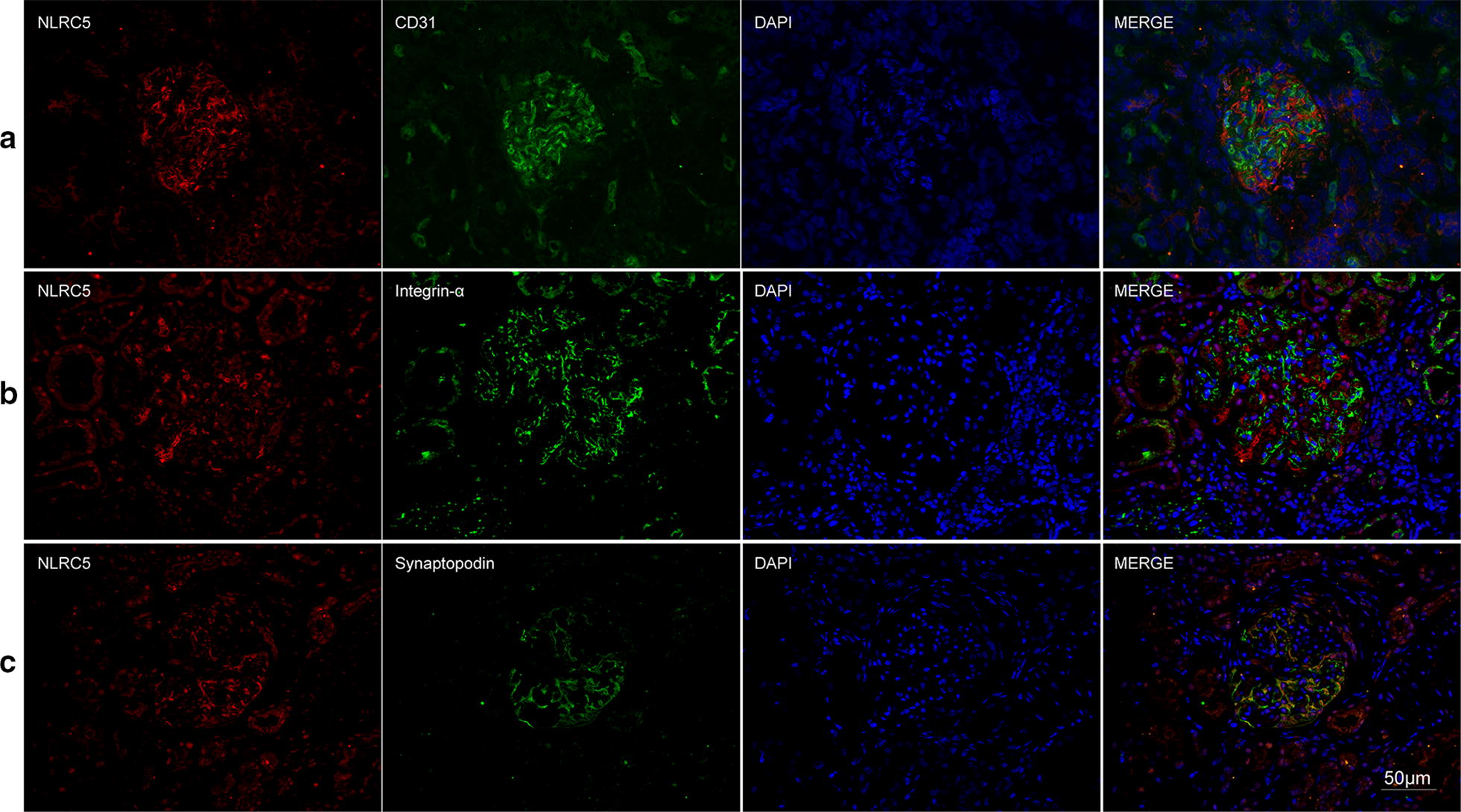
Double immunofluorescence staining of NLRC5 and glomerular intrinsic cells in AAV patients. **a** Co-localization of NLRC5 (red) and CD31 (green). **b** Co-localization of NLRC5 (red) and integrin-α (green). **c** Co-localization of NLRC5 (red) and synaptopodin (green). Scale bar = 50 μm in the bottom right

**Fig. 6:**
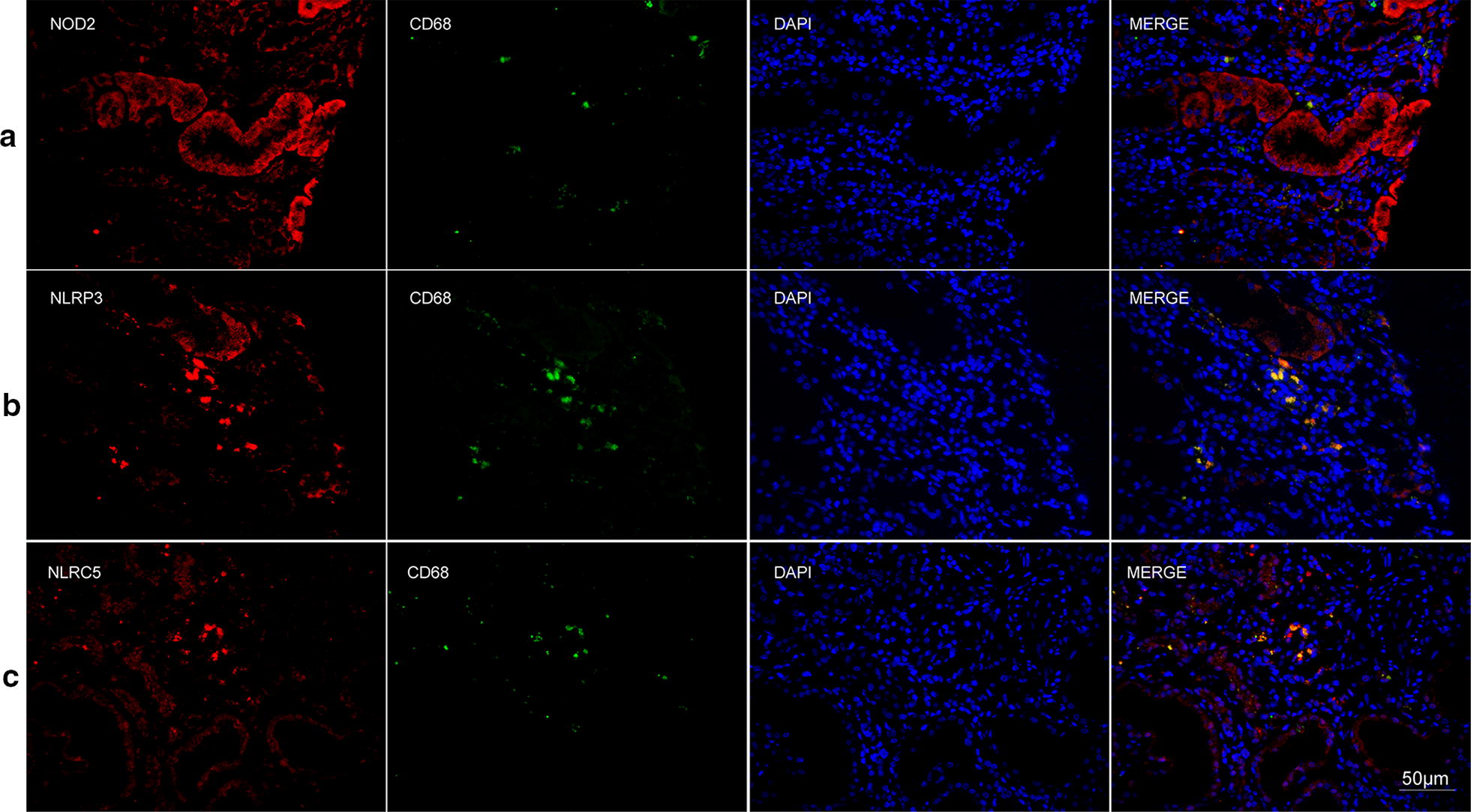
Double immunofluorescence staining of NLRs and infiltrating cells in AAV patients. **a** Co-localization of NOD2 (red) and CD68 (green). **b** Co-localization of NLRP3 (red) and CD68 (green). **c** Co-localization of NLRC5 (red) and CD68 (green). Scale bar = 50 μm in the bottom right

The immunohistochemical assay showed that three NLRs were universally expressed in glomeruli and tubulointerstitium in AAV patients. To further investigate locations of NLRs, we performed double immunofluorescence staining for NLRs and markers of various cell types (Figs. 3, 4, 5, 6). Among the renal intrinsic cells, NOD2, NLRP3 and NLRC5 were predominantly expressed in podocytes and sparsely expressed in glomerular endothelial or mesangial cells. The staining of three NLRs was also detected in infiltrating monocytes/macrophages specifically marked with CD68. Collectively, three NLRs co-localized with podocytes and infiltrating inflammatory cells.

### The gene expression of NLRs in human podocytes

**Fig. 7:**
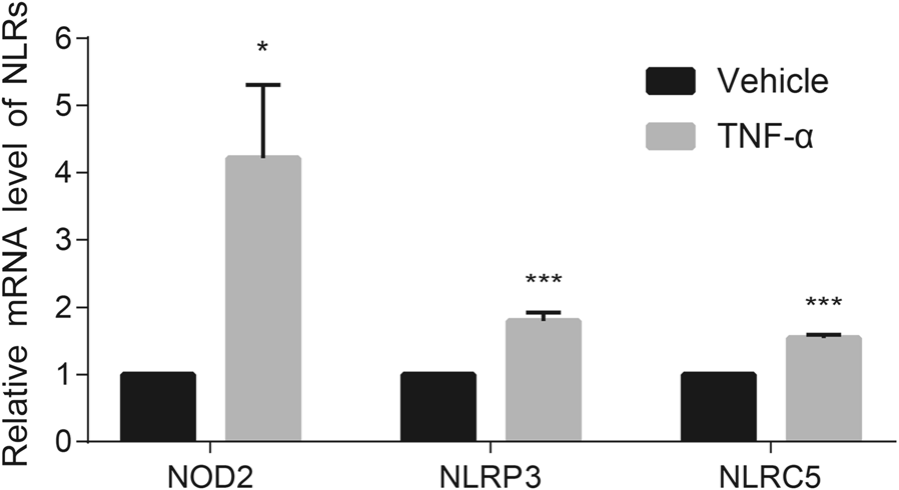
The mRNA expression of three NLRs in human podocytes. The mRNA expression of NOD2, NLRP3 and NLRC5 in cultured human podocytes was elevated upon TNF-α stimulation. Bars represent mean ± standard error of repeated measurements of four independent experiments. *ns* no significance. *P < 0.05, **P < 0.01, ***P < 0.001 compared with vehicle treatment

In order to validate the expression results of immunohistochemical staining, we employed quantitative polymerase chain reaction to determine the expression of three NLRs on mRNA levels. Given the formation of crescents in AAV was associated with podocyte injury [33] and NLRs mainly co-localized with podocyte in glomeruli, we employed human podocytes for the functional study in vitro. Consistent with the results of immunohistochemical staining, the expression of NOD2, NLRP3 and NLRC5 were upregulated upon TNF-α stimulation (Fig. 7).

### Correlation analysis of NLRs staining and clinicopathological data

**Fig. 8:**
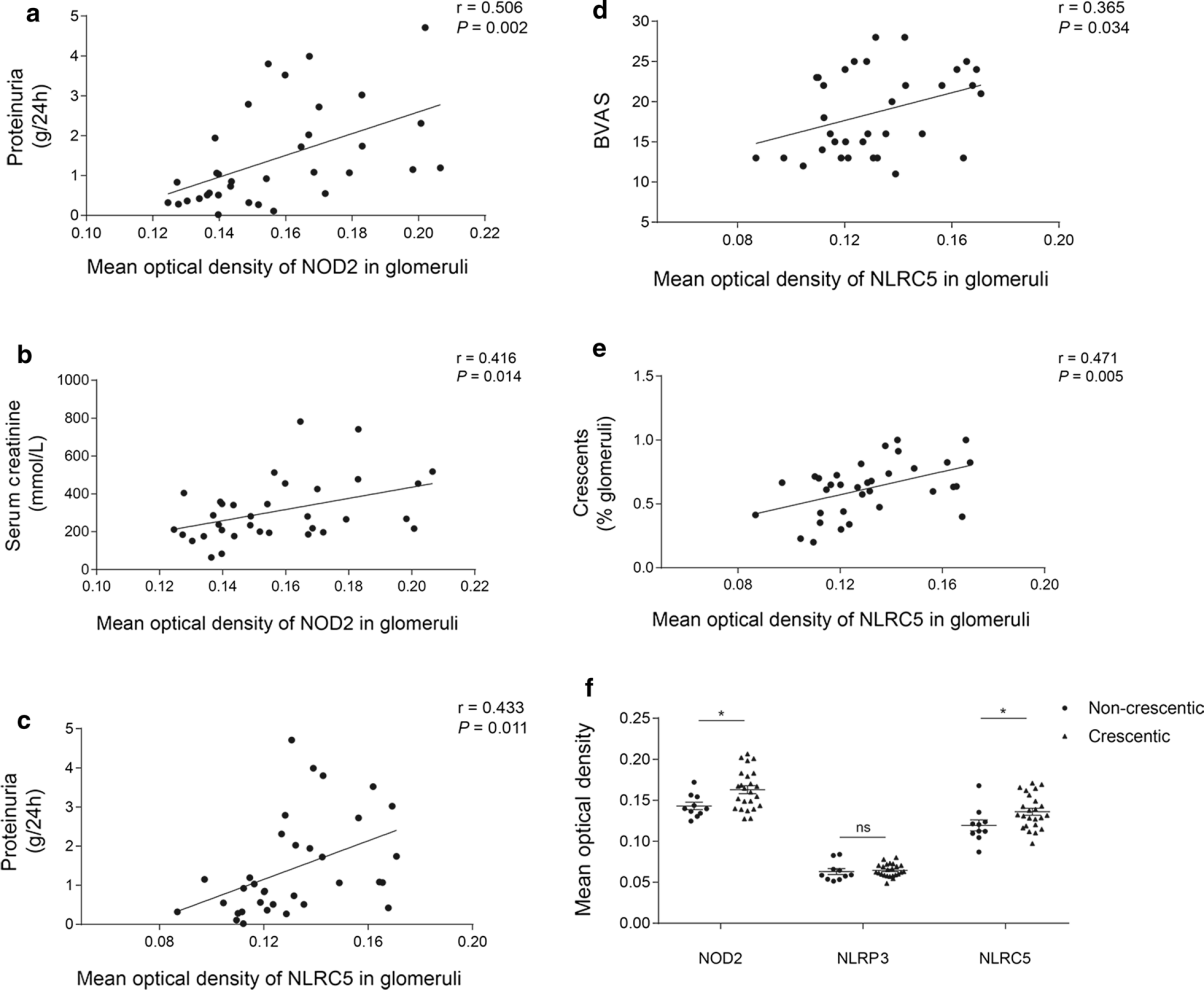
Correlations of NLRs and clinicopathological data in AAV patients.** a**, **b** The mean optical density of NOD2 in glomeruli correlated with proteinuria and serum creatinine. **c**–**e** The mean optical density of NLRC5 in glomeruli correlated with proteinuria, BVAS and proportion of crescents. **f** Glomerular expression of three NLRs in different categories of Berden classification. Data were shown as mean ± standard error. *ns* no significance. *P < 0.05

Then, we made correlation analysis between the expression of NLRs and clinicopathological parameters in AAV patients (Fig. 8). For NOD2, the mean optical density in glomeruli correlated with proteinuria level and serum creatinine at renal biopsy (r = 0.506, *P* = 0.002; r = 0.416, *P* = 0.014). For NLRP3, there was no significant correlation between the expression and clinicopathological data. For NLRC5, the mean optical density in glomeruli correlated with proteinuria level, BVAS and the proportion of crescents in renal specimen (r = 0.433, *P* = 0.011; r = 0.365, *P* = 0.034; r = 0.471, *P* = 0.005, respectively). As for the classification scheme proposed by Berden et al., the mean optical density of NOD2 and NLRC5 in glomeruli was significantly higher in crescentic class than that in non-crescentic class (0.163 ± 0.005 *vs.* 0.143 ± 0.004, *P* = 0.021; 0.136 ± 0.004 *vs.* 0.119 ± 0.007, *P* = 0.041) (Fig. 8).

## Discussion

As phyla conserved sensors of cytosolic invading pathogens and harmful molecules associated with cell stress, NLRs play an important role in immune response through canonical and noncanonical manners [7, 11]. However, the expression of NLRs and their clinical significance in AAV are not fully clear yet.

In this study, we found upregulated expression of NOD2 and NLRC5 in renal specimen of AAV patients and in podocytes upon TNF-α stimulation in vitro. The glomerular expression of NOD2 and NLRC5 in crescentic class was significantly higher than that in non-crescentic class. Further correlation analysis revealed significant correlation between these two NLRs and severity of renal involvement, including proteinuria level, serum creatinine, and the proportion of crescents in renal specimen. As for NOD2, Uehara et al. reported that PR3-ANCA endowed kidney epithelial cells and monocytes the capability to produce proinflammatory cytokine like IL-6, IL-8, MCP-1 and TNF-α upon the stimulation of pathogen-associated molecular pattern such as muramyl dipeptide (MDP, a typical agonist to NOD2) [13, 14]. NOD2 could exert harmful impact on acute kidney injury induced by ischemia reperfusion or systemic administration of lipopolysaccharide/peptidoglycan, and could promote renal injury in diabetic nephropathy in canonical and noncanonical manners [11, 15, 24, 34]. As the largest NLR, NLRC5 participated in the pathogenesis of renal fibrosis, ischemia–reperfusion renal injury and diabetic nephropathy via modulating various signaling pathways including NF-κB and TGF-β/Smad [20–23]. The deficiency of NOD2 or NLRC5 in mice also showed significant kidney protection from acute or chronic injury in above-mentioned diseases. Collectively, the NOD2 and NLRC5 showed proinflammatory effects in some renal diseases, and our results revealed a potential association between the NLRs and the development of AAV.

As for NLRP3, Tashiro et al. reported that in patients with MPO-ANCA associated glomerulonephritis, NLRP3 protein was detected in macrophages and the severe infiltrated area but was absent or only faintly expressed in glomeruli [19], which was consistent with our results. No significant correlation between the expression of NLRP3 and the severity of renal injury was found in our study. It indicated that the renal injury in AAV was independent of the direct effect of NLRP3-inflammasome, like the situation in anti-GBM disease [35].

The current study also found that all these three NLRs co-localized with podocytes and monocytes/macrophages. After TNF-α treatment, the mRNA expression of NLRs was elevated in cultured human podocytes. Our previous study reported that the structural damage and detachment of podocytes occurred in patients with ANCA-associated glomerulonephritis, and podocyte detachment was an independent predictor of poor renal outcomes [33]. Consistent with our findings, Hewins et al. also demonstrated that podocytes were the predominant glomerular IL-18 (a representative terminal inflammatory factor produced from the activation of inflammasomes) positive cell type in AAV patients [36]. Noronha et al. found evident terminal inflammatory IL-1β staining in the interstitium and perivascular site in ANCA-positive glomerulonephritis, but didn’t find elevated IL-1β in most plasma samples, which suggested the contribution of intrinsic renal cells to vasculitis [37].

## Conclusions

In AAV patients, the expression of NOD2, NLRP3 and NLRC5 was upregulated in kidneys; the expression of NOD2 and NLRC5 correlated with the severity of renal injury in AAV. These findings might help to understand the pathogenesis of AAV and inspire potential therapeutic targets in further studies.

## Data Availability

All data generated or analysed during this study are included in this published article.

## Abbreviations

ANCA: anti-neutrophil cytoplasmic antibody
AAV: ANCA-associated vasculitis
NLR: nucleotide-binding oligomerization domain-like receptor
NOD2: nucleotide-binding oligomerization domain containing protein 2
NLRP3: NOD-like receptor family pyrin domain containing 3
NLRC5: NOD-like receptor family CARD domain containing 5
MPO: myeloperoxidase
PR3: proteinase 3
ESR: erythrocyte sedimentation rate
Scr: serum creatinine
eGFR: estimated glomerular filtration rate
BVAS: Birmingham Vasculitis Activity Scores
ENT: ear, nose and throat
